# Four-Dimensional Printing of β-Tricalcium Phosphate-Modified Shape Memory Polymers for Bone Scaffolds in Osteochondral Regeneration

**DOI:** 10.3390/ma18020306

**Published:** 2025-01-11

**Authors:** Izabella Rajzer, Anna Kurowska, Jarosław Janusz, Maksymilian Maślanka, Adam Jabłoński, Piotr Szczygieł, Janusz Fabia, Roman Novotný, Wojciech Piekarczyk, Magdalena Ziąbka, Jana Frankova

**Affiliations:** 1 Department of Mechanical Engineering Fundamentals, Faculty of Mechanical Engineering and Computer Science, University of Bielsko-Biala, 43-300 Bielsko-Biała, Poland; 2 Faculty of Materials, Civil and Environmental Engineering, University of Bielsko-Biala, 43-300 Bielsko-Biała, Poland; 3 Department of Medical Chemistry and Biochemistry, Faculty of Medicine and Dentistry, Palacký University Olomouc, 779 00 Olomouc, Czech Republic; 4 Department of Glass Technology and Amorphous Coatings, Faculty of Materials Science and Ceramics, AGH University of Krakow, 30-059 Kraków, Poland; 5 Department of Ceramics and Refractories, Faculty of Materials Science and Ceramics, AGH University of Krakow, 30-059 Kraków, Poland

**Keywords:** 4D printing, shape memory polymers, scaffolds, bone tissue engineering

## Abstract

The use of scaffolds for osteochondral tissue regeneration requires an appropriate selection of materials and manufacturing techniques that provide the basis for supporting both cartilage and bone tissue formation. As scaffolds are designed to replicate a part of the replaced tissue and ensure cell growth and differentiation, implantable materials have to meet various biological requirements, e.g., biocompatibility, biodegradability, and mechanical properties. Osteoconductive materials such as tricalcium phosphate ceramics and some biodegradable polymers appear to be a perfect choice. The present work evaluates the structural, mechanical, thermal, and functional properties of a shape memory terpolymer modified with β-tricalcium phosphate (β-TCP). A new approach is using the developed materials for 4D printing, with a particular focus on its applicability in manufacturing medical implants. In this study, the manufacturing parameters of the scaffold components were developed. The scaffolds were examined via scanning electron microscopy with energy dispersive spectroscopy (SEM-EDS), Fourier-transform infrared spectroscopy (FTIR), differential scanning calorimetry (DSC), and mechanical testing. The cytotoxicity result was obtained with an MTT assay, and the alkaline phosphatase (ALP) activity was measured. The structural and microstructural investigations confirmed the integration of β-TCP into the filament matrix and scaffolds. Thermal stability was enhanced as β-TCP delayed depolymerization of the polymer matrix. The shape memory studies demonstrated effective recovery. The in vitro cell culture studies revealed the significantly increased cell viability and alkaline phosphatase (ALP) activity of the β-TCP-modified terpolymer after 3 weeks. The developed terpolymer can be tailored for applications in which partial shape recovery is acceptable, such as bone scaffolds.

## 1. Introduction

Since cartilage and subchondral bone have different biological properties, treating osteochondral defects remains a significant challenge [1]. The osteochondral interface is particularly difficult to regenerate due to its limited regenerative capacity and complex stratified architecture [2]. The osteochondral unit is a unique tissue that includes bone, cartilage, and transitional layers, each with gradated mechanical and biological properties [3]. The fabrication of new implants that guide osteochondral tissue regeneration, with properties and functions that gradually change to match the injured tissue, remains a major issue.

Additive manufacturing, particularly 3D printing, has gained significant interest in recent decades as a fabrication platform for implants [4]. This technology involves depositing materials layer by layer using computer-aided controls, resulting in high-precision 3D geometries with the desired spatial arrangement [5]. However, currently produced 3D-printed implants are still unable to closely mimic the native tissue, especially the biological properties of osteochondral tissue [6]. Furthermore, implants must often achieve a predefined shape to allow for delivery via minimally invasive methods.

Four-dimensional printing offers a solution that meets the desired requirements. It combines 3D printing with the fourth dimension—time [7]. While 3D printing creates static objects layer by layer based on a digital model, 4D printing involves the creation of objects that can change shape, function, or behavior over time in response to environmental factors such as heat, moisture, light, or other stimuli. This novel technology employs smart materials capable of expanding, flexing, and/or deforming in response to specific stimuli. An example is shape memory polymers (SMPs), which can change shape when exposed to external stimuli, such as temperature. SMPs are capable of deforming and recovering their original shape, which makes them highly suited for osteochondral regeneration [8]. SMPs used in biomedical applications must comply with several critical criteria, including biocompatibility, sterilizability, ease of processing, and excellent mechanical properties. Additionally, their switching temperature should be close to or slightly above body temperature. Thermoplastic bioresorbable shape memory polymers are particularly promising for such applications. These materials, which are processed through 3D printing, can be used to create self-expanding stents, self-clamping devices, loops, and scaffolds for treating large bone defects [9]. Their use has the potential to introduce or enhance numerous innovative surgical techniques.

Polyglycolide and its copolymers with lactide and caprolactone have been increasingly used in medical applications due to their biocompatibility and favorable mechanical properties [10]. These materials are characterized by safe biodegradation, commercial availability, degradability in physiological environments, and the ability to support healing [11]. Moreover, they can be combined with other biomaterials that exhibit crystal structures and chemical properties similar to the inorganic components of bone tissue, e.g., materials based on tricalcium phosphate (TCP) [12,13]. β-TCP is widely recognized in reconstructive surgeries for its osteoconductive and osteoinductive properties, which facilitate bone regeneration. However, its bioresorbability is relatively limited in comparison to other materials, solidifying its role as a key substance in bone tissue engineering [14,15]. While various stimuli-responsive microstructures have been developed, 4D printing technology remains in its early stages and still requires substantial advancements in material innovation.

The goal of this study was to investigate the β-TCP modification impact on the structural, mechanical, thermal, and functional properties of a shape memory terpolymer. The research focused on evaluating β-TCP integration and distribution within the polymer matrix, its effect on the material’s crystallinity, thermal stability, and shape memory behavior, as well as its suitability for various processing methods, such as injection molding and 3D printing. Ultimately, this study aimed to assess the potential of this modified terpolymer for advanced applications via 4D printing and, thus, the development of medical implants endowed with enhanced functionality and biocompatibility.

## 2. Materials and Methods

### 2.1. Materials

A segmental terpolymer consisting of L-lactide, ε-caprolactone, and glycolide (L-LA/GL/ε-CL 69/16/15) was prepared through ring-opening polymerization at the Centre for Polymer Materials of the Polish Academy of Sciences using zirconium acetylacetonate as a biocompatible initiator [16,17]. The terpolymer was mechanically mixed with 0.5% wt. β-tricalcium phosphate particles (β-TCP, Merc, Warszawa, Poland) and homogenized at 190 °C. The blending process was carried out by rolling alloys of the plastic material, from which disks with diameters of 0.6 cm and 1.2 cm were cut, corresponding to the well diameters of the cell culture plate. The remaining blend was then processed into granules for the injection molding process. For comparison, pure terpolymer samples in the form of disks (BL) were also prepared.

### 2.2. Scaffold Fabrication

Injection molding was performed using a Babyplast 6/10P machine (Rambaldi, Molteno, Italy). The terpolymer was fabricated in the form of filament sticks using a custom-designed mold that was described previously [18]. Additionally, tensile test specimens were produced using a mold designed for manufacturing dog bone-shaped samples. The injection molding parameters for both the dog bone-shaped samples and the filament sticks are presented in Table 1.

**Figure 1:**
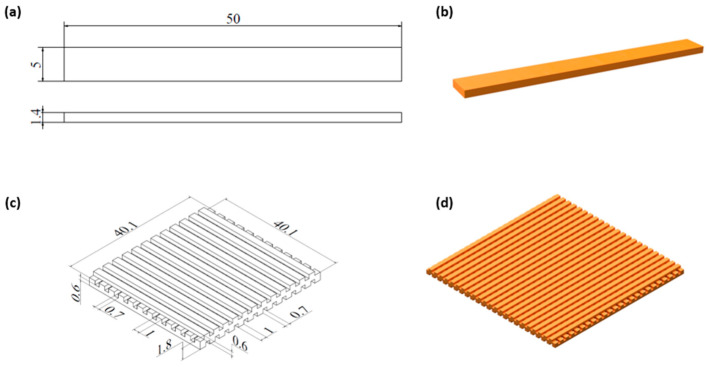
Models of cuboid sample (**a**,**b**) and lattice structure with dimensions (mm) (**c**,**d**).

Computer-aided design (CAD) software (Autodesk Inventor Professional, 2023, Autodesk, Inc., San Rafael, CA, USA, version number of the software: v27.50.44600.0000) was used to design 3D samples characterized by a cuboid shape (Figure 1a,b) and a lattice structure. The model of a lattice consisted of three layers made of bars with a rectangular cross-section (1 × 0.6 mm) spaced 0.7 mm apart; the adjacent layers were perpendicular to each other. The obtained lattice structure had cuboid-shaped pores (Figure 1c,d). The filament sticks of 1.75 mm in diameter were extruded at 150 °C with a commercial 3D printer (Prusa i3 MK3S+, Prague, Czech Republic) in the FFF/FDM technology. The print bed temperature was set to 40 °C, cooling was turned on, the nozzle diameter was 0.8 mm, the layer thickness equaled 0.3 mm, and the infill was 100%.

### 2.3. Characterization

#### 2.3.1. Mechanical Testing

The mechanical properties of the pure terpolymer were determined through tensile and ultrasonic testing on the dog bone-shaped specimens. Polycaprolactone samples (Mn 80 kDa, Merck, Warszawa, Poland) were used as a reference. The injection molding parameters are presented in Table 1. The ultrasonic test of pure terpolymer samples was conducted using a CT-3 materials tester (Unipan-Ultrasonics, Warsaw, Poland). Longitudinal wave velocities were determined using the through-transmission method, with 1 MHz transducers and an adhesive tape serving as the coupling medium. The average velocities and standard deviations were calculated. Tensile tests were performed on a universal testing machine (Hegewald & Peschke, Nossen, Germany). Young’s modulus was determined within a strain range of 0.05% to 0.25% at a testing speed of 1 mm/min, while the crosshead speed was increased to 10 mm/min for the remaining test. Six specimens, each approximately 2.08 mm thick and 2.12 mm wide, were analyzed.

#### 2.3.2. Morphological Observation

Analyses of the morphology, structural uniformity, and potential surface defects of the obtained disks, filaments, and scaffolds were performed using an Opta-Tech stereomicroscope (Opta-Tech, Warszawa, Poland) equipped with a CMOS 3 camera and OptaView 7 software.

The evaluation of the samples’ microstructures and scaffold architectures, as well as the morphology of cells seeded on the materials, was performed using the Apreo 2S low vac high-resolution scanning electron microscope from ThermoFisher Scientific. Prior to their imaging, all the samples were coated with a thin conducting layer of carbon (10 nm) and observed in low vacuum (50 Pa) conditions with LVD and CBS detectors at an accelerating voltage of 10 kV. The pore linear dimensions of scaffolds were calculated from the SEM pictures with the Image Line measurement program. The elemental content of active modifiers incorporated in the sticks and the blends was examined using an EDAX Octane Elite energy dispersive X-ray spectroscopy (EDS/EDX) system with Octane Elite silicon drift detectors (SDDs) operated with APEX™ Advanced 2022 software, version 2.5.1001.001. The qualitative analysis was performed using a standardless method.

#### 2.3.3. Fourier-Transform Infrared Spectroscopy

ATR-FTIR spectra were obtained over a wavenumber range of 350–4000 cm^−1^ using a Thermo Scientific Nicolet iS5 FT-IR Spectrometer (Thermo Fisher Scientific, Warszawa, Poland) equipped with an iD7 ATR module and a diamond crystal for attenuated total reflectance (ATR). The spectra were recorded with 32 scans at a resolution of 4 cm^−1^.

#### 2.3.4. Thermal Analysis

Differential scanning calorimetry (DSC) measurements were carried out with an analytical system (TA Instruments, New Castle, DE, USA) equipped with an MDSC Calorimeter 2920 and a refrigerated cooling system. The samples were heated at a rate of 20 °C/min from −30 °C to about 180 °C and, in selected cases, up to 225 °C, followed by cooling at the same rate. The experiments were performed in a nitrogen atmosphere (purge flow rate: 40 cm³/min) using standard aluminum pans. The weight of the examined samples was about 5 mg. The registration sensitivity was 0.2 μW. An analysis of the DSC curves, including the determination of enthalpies and characteristic transition temperatures, was carried out using Universal V4.5A software provided by TA Instruments. Thermogravimetric (TGA) investigations were performed using a TA Instruments Q500 Thermogravimetric Analyzer. The measurements were performed at a temperature ranging from 30 to 650 °C with a heating rate of 20 °/min in a nitrogen atmosphere (flow 60 mL/min). The TGA data were analyzed using TA Universal V4.5 software.

#### 2.3.5. Shape Memory Analysis

**Figure 2:**
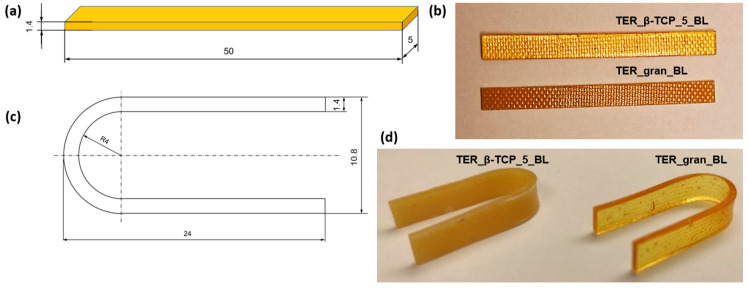
Samples for shape memory tests: (**a**,**b**) initial shape and (**c**,**d**) temporary shape.

To study shape memory, samples with dimensions of 50 × 5 × 1.4 mm (original shape shown in Figure 2a,b) were prepared. Plates with a thickness of 1.4 ± 0.1 mm were produced by rolling alloys of the investigated materials, and the samples were subsequently cut with a mechanical press. The samples were shaped into a temporary “U” shape (Figure 2c,d) in distilled water at a temperature of 45 °C and then cooled in water at 10 °C for 20 min. Prior to testing, the samples were stored at room temperature of approximately 20 °C.

The shape memory test (ability to return to the original shape) was conducted by observing and recording a video of the deformation process of the samples after immersion in distilled water at 37 ± 0.3 °C. The tests were performed on 5 samples made from the base material TER_GRAN_BL and 5 samples made from the β-TCP modified material (TER_BTCP_5_BL).

### 2.4. Cell Culture Studies

#### 2.4.1. Cultivation of Saos-2

Saos-2 cells were maintained in McCoy’s 5A medium supplemented with 10% FBS and penicillin-streptomycin at 37 °C in a 5% CO_2_ incubator. When the cells reached approximately 80% confluence, they were passaged. The culture medium was removed, and the cells were washed twice with 15–20 mL of 10% PBS. The trypsin/EDTA solution was then added, and the flask was incubated at 37 °C with 5% CO_2_ for 2–3 min until the cells detached. Following the detachment, the fresh culture medium containing serum was added to inhibit the trypsin activity. The cell suspension was centrifuged at 1000 rpm for 3 min at room temperature. The supernatant was discarded, and the cell pellet was resuspended in 5 mL of fresh medium. The suspension was either transferred to new culture flasks or counted and plated for further culture.

#### 2.4.2. Sterilization of Materials

The materials used in the tests underwent sterilization via immersion in 70% ethanol for 24 h, followed by UV irradiation for 20 min on both sides.

#### 2.4.3. MTT Assay

Experiments were performed using both the unmodified terpolymer and the terpolymer modified with β-TCP. The materials in the form of disks were placed in 96-well plates, and a cell suspension (1 × 10^4^ cells/mL, 0.2 mL per well) was added to each well. The plates were incubated for 1 day, 1 week, 2 weeks, and 3 weeks at 37 °C in 5% CO_2_. After the incubation period, the medium was removed, and each well was rinsed with 10% PBS. An MTT solution (serum-free culture medium: MTT, 10:1) was then added to each well, and the plates were incubated for 3 h at 37 °C in 5% CO_2_. Following the incubation, the medium was discarded, and DMSO with NH_3_ was added to each well. The DMSO-NH3 mixture was transferred to a new plate, and the absorbance was measured at 550 nm.

#### 2.4.4. Determination of Alkaline Phosphatase (ALP)

Saos-2 cells were seeded onto the tested materials in 24-well culture plates at a concentration of 2 × 10^4^ cells/mL and incubated for 24 and 72 h. After the incubation, the cells were lysed in PBS containing 0.2% Triton X-100 (PBS-T), and the alkaline phosphatase (ALP) activity was assessed by measuring the conversion of colorless p-nitrophenol phosphate to yellow p-nitrophenol at alkaline pH. The p-nitrophenol production was monitored spectrophotometrically at 405 nm and quantified via comparison with a standard curve using p-nitrophenol dissolved in PBS-T. The ALP levels were normalized to the protein content, which was determined using the Bradford method with bovine serum albumin as the standard [19].

#### 2.4.5. Fluorescence Staining

The materials were sterilized and placed into a 24-well plate, where Saos-2 cells were seeded at a final concentration of 1 × 10^5^ cells/mL (0.8 mL per well). After 24 h, 1 week, or 3 weeks, the medium was removed, and the cells were washed with PBS. The cells were then stained with 0.01% acridine orange (0.3 mL per well) for 1 min in the dark. Following this, the cells were washed 10 to 20 times with PBS. Finally, the materials were transferred to microscope slides and examined using a fluorescence microscope.

#### 2.4.6. Biocompatibility Evaluation

SEM microscopy was used to evaluate biocompatibility. Cells were seeded on pre-wetted and UV-irradiated samples at a final concentration of 0.16 × 10^5^ cells per well and allowed to adhere for 1 week. The cells were fixed by rinsing them three times with PBS buffer before they were treated with 2.5% glutaraldehyde for 30 min. After fixation, the samples underwent dehydration using ethanol solutions of increasing concentrations: 25%, 40%, 60%, 80%, 90%, and 100%, with each step lasting 15 min. Following the final 100% ethanol step, the samples were incubated for 10 min with hexamethyldisilazane (HMDS). Finally, the prepared samples were observed using a scanning electron microscope.

#### 2.4.7. Statistical Analysis

Cell viability results were expressed as the mean ± standard deviation. The statistical analysis was performed using GraphPad Prism 8. The *t*-test was employed to compare the cell viability value on the modified materials with the one on TER_gran_BL. The comparisons were made between the samples incubated for the same time period. A *p*-value of 0.05 was considered statistically significant.

## 3. Results

### 3.1. Morphological Characterization of β-TCP-Modified Disks, Sticks, and Scaffolds

**Figure 3:**
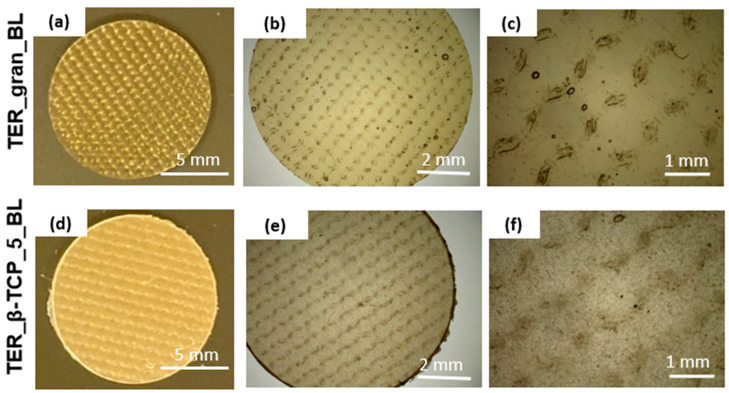
Stereoscopic microscope images of the terpolymer alloys at various magnifications, (**a**–**c**) pure terpolymer in the form of a disk, (**d**–**f**) terpolymer with 5wt.% β-TCP.

To evaluate the architectural design of the terpolymer blends, the samples (disks) were analyzed using stereoscopic microscopy at varying magnifications (Figure 3). These images revealed the detailed surface texture resulting from the manufacturing process. In the samples modified with β-TCP, distinct powder-derived inclusions were observed. These inclusions were uniformly distributed across the surface, highlighting the effective integration of β-TCP into the matrix.

**Figure 4:**
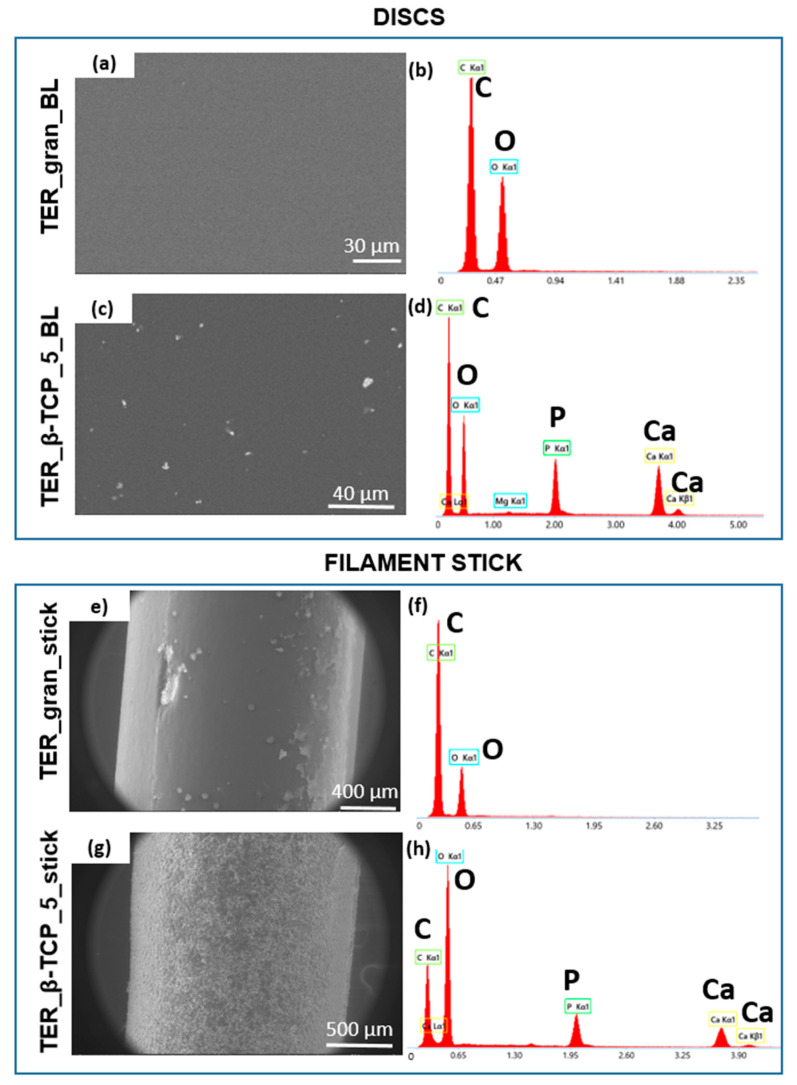
SEM images with EDS analysis of disks (**a**–**d**) and filament sticks (**e**–**h**) of TER_gran and TER_β-TCP_5 samples.

Scanning electron microscopy provided additional details on the surface characteristics (Figure 4). While the pure terpolymer samples exhibited a smooth and homogeneous surface, the β-TCP-modified samples displayed visible agglomerates, confirming the additive presence in the matrix. The agglomerate distribution indicated successful blending and β-TCP dispersion within the polymer.

The EDS method was used to verify the samples’ chemical compositions. This analysis confirmed the β-TCP incorporation into the polymer matrix, as evidenced by the presence of chemical elements associated with the additives, i.e., calcium and phosphorus. The modified material was also examined in different forms, including filaments and scaffolds produced via injection molding and 3D printing techniques.

**Figure 5:**
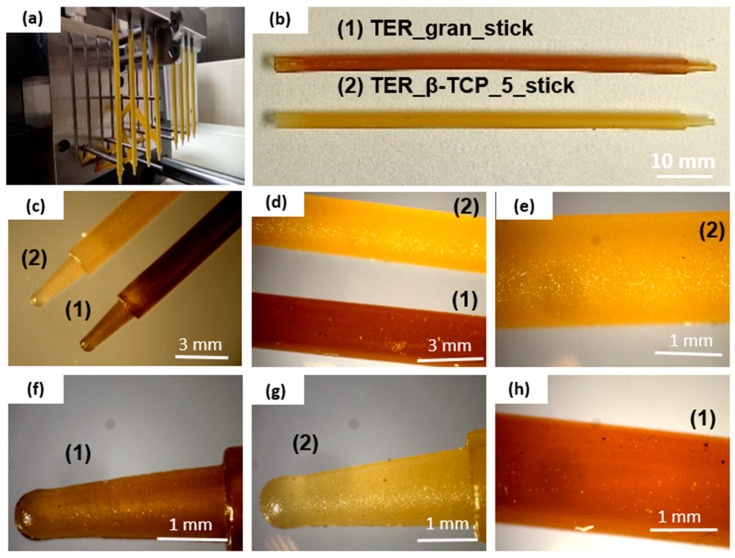
Stereoscopic microscope images of filament sticks at various magnifications, (**a**) filament sticks directly after injection molding process, (**b**) pure terpolymer (1) stick, terpolymer with 5 wt.% β-TCP (2) stick, (**c**,**f**,**g**) arrowhead of two types of sticks, (**d**,**e**,**h**) surface of two types of sticks.

**Figure 6:**
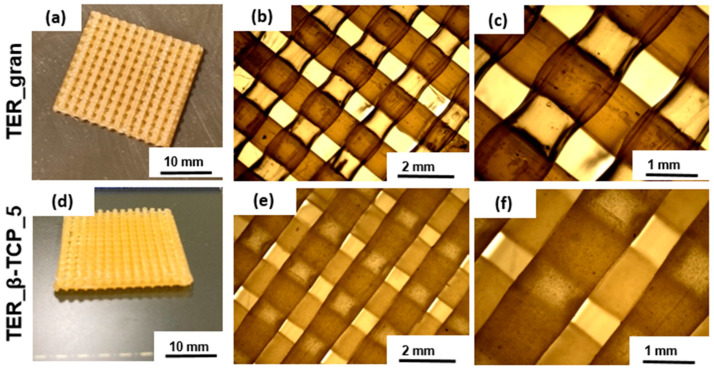
Stereoscopic microscope images of the scaffolds at various magnifications, (**a**–**c**) pure terpolymer, (**d**–**f**) terpolymer with 5wt.% β-TCP.

**Figure 7:**
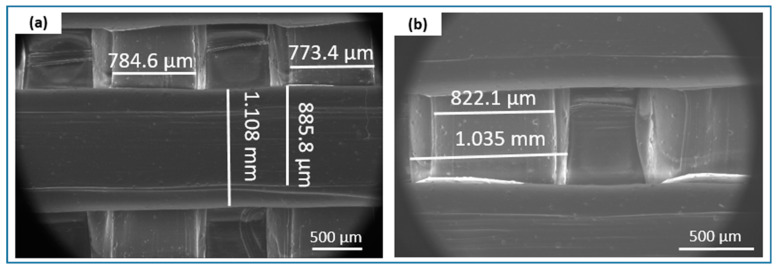
SEM images of terpolymer scaffold modified with β-TCP: (**a**) central upper path with a linear size of 1.1 mm is visible; (**b**) in the depth, the lower path with a linear size of 1.0 is visible.

The microscopic studies indicated that both the filament sticks and the 3D scaffolds were successfully fabricated. The mold shape was accurately reproduced, resulting in high-quality sticks (Figure 5). The analysis confirmed that both types of printed scaffolds exhibited a highly porous microstructure with interconnected pores (Figure 6). The results consistently demonstrated the successful incorporation and uniform distribution of the β-TCP powder within the filament sticks and the resulting scaffolds. The SEM images of the terpolymer scaffold modified with β-TCP (Figure 7) revealed numerous powder agglomerates dispersed within the structure, suggesting the partial inhomogeneity of the modifier distribution. The lattice model, consisting of three layers of bars, was visible in the SEM micrographs. The obtained lattice structure was characterized by cuboid-shaped pores, which aligned with the intended geometric design of the scaffold. The 3D printing process ensured a highly interconnected pore network.

### 3.2. Mechanical Properties of Pure Terpolymer

Table 2 presents the mechanical properties of the pure terpolymer in comparison with polycaprolactone—another polymer frequently used to produce filaments for 3D printing [17]. These parameters include tensile strength, Young’s modulus, strain at break, and longitudinal wave velocity, with the values given as mean ± standard deviation. The measured mechanical properties proved the terpolymer to be endowed with relatively high values of tensile strength and Young’s modulus compared to polycaprolactone. The terpolymer showed more limited elongation, while polycaprolactone exhibited a much higher strain at break, proving it was more flexible and capable of undergoing larger deformations before fracture. The terpolymer specimens exhibited a 13% higher wave velocity compared to the PCL samples, which directly translated into Young’s modulus, as confirmed by the data from the tensile testing machine. Although a direct comparison of the mechanical properties of the terpolymer with and without ceramic particle inclusion was not performed in this study, future studies could include such a comparison to assess the impact of ceramic inclusion on the material’s mechanical strength.

### 3.3. FTIR Spectral Analysis of Terpolymer and β-TCP Composites

**Figure 8:**
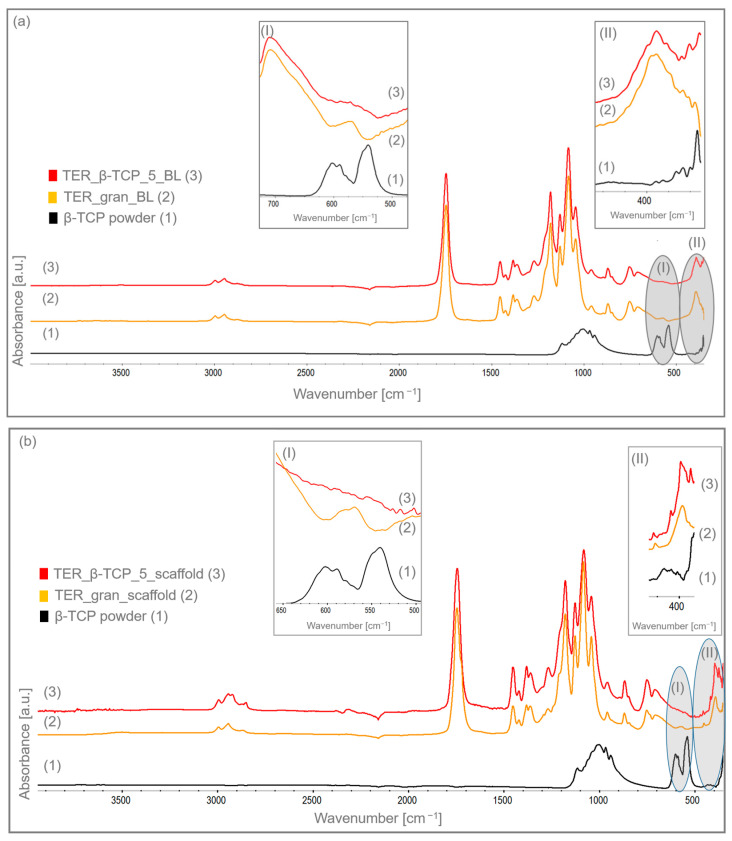
FTIR spectra of (**a**) disk samples (BL) and (**b**) scaffolds: (1) β-TCP powder; (2) terpolymer modified with 5% β-TCP; and (3) pure terpolymer, including magnified wavenumber ranges of (I) 500–650 cm^−1^ and (II) 350–450 cm^−1^.

The infrared spectrum (Figure 8a,b) revealed characteristic bands indicative of specific functional groups in the monomers. The vibrations related to the C-H stretching in CH and CH_3_ groups appeared at 2995 cm^−1^ and 2945 cm^−1^, respectively. A band at 1745 cm^−1^ corresponded to the carbonyl (C=O) stretching in ester bonds, i.e., a characteristic property of L-lactide, glycolide, and caprolactone monomers. The bending vibrations of C-H in -CH_2_ groups, mainly from caprolactone and partly from glycolide segments, were detected at 1452 cm^−1^ and 1425 cm^−1^. Similarly, the C-H bending vibrations of -CH_3_ groups specific to L-lactide manifested at 1382 cm^−1^ and 1364 cm^−1^. The stretching of C-O and C-O-C bonds in ester linkages was marked by a prominent band at 1180 cm^−1^, which is a defining trait of the terpolymer structure. The additional bands, located at 1128 cm^−1^, 1084 cm^−1^, and 1044 cm^−1^, were associated with the C-O and C-O-C stretching vibrations within ester bonds of caprolactone. These bands confirmed the incorporation of functional groups derived from all three monomers in the terpolymer [20,21]. β-TCP exhibited characteristic bands at 1003 cm^−1^ and 1116 cm^−1^ corresponding to the asymmetric P–O stretching vibrations. The symmetric stretching vibration of the P–O group was observed at 968 cm^−1^ and 942 cm^−1^. The bands around 602 cm^−1^ and 540 cm^−1^ were associated with the bending vibrations of phosphate groups.

There was an absence of peaks at 602 cm^−1^ and 540 cm^−1^, which is typically associated with biological tricalcium phosphate (β-TCP) [22]. Probably, when the terpolymer and β-TCP exhibited absorption features in the same spectral region, their overlapping vibrational modes attenuated the individual peak intensities. As a result of this interaction, the distinct peaks merged into a broader, more diffused band. Consequently, the FTIR spectrum of both the modified disks and scaffolds appeared less resolved, with a more continuous or flattened profile where the characteristic β-TCP peaks would typically have been observed. The lack of these bands in the modified terpolymer indicated the β-TCP incorporation into the polymer, thus confirming the successful modification.

### 3.4. Thermal Analysis: DSC and TGA of Terpolymer and β-TCP Modified Materials

**Figure 9:**
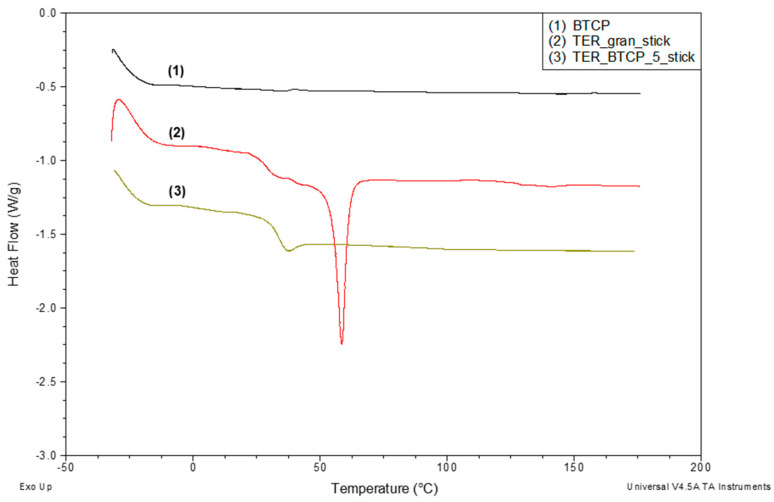
DSC curves for β-TCP modifier and sticks molded from investigated terpolymer (without and with 5% β-TCP) recorded over a temperature range of −30 °C to approx. 180 °C during heating at 20 °C/min rate under nitrogen flow of 40 mL/min.

Figure 9 presents the DSC curves recorded for three materials: the base shape memory terpolymer (curve 2), the terpolymer modified with 5% β-TCP (curve 3), and the pure modifier β-TCP (curve 1). The DSC curve for the pure terpolymer (30–180 °C) demonstrated its semicrystalline structure, as evidenced by the glass transition (characteristic shift) and a distinct melting peak.

Upon incorporating 5% β-TCP into the terpolymer, the melting peak disappeared entirely, indicating the crystalline phase elimination from the terpolymer structure. Additionally, the DSC curve for the modified terpolymer showed an endothermic enthalpy relaxation effect (apparent melting) associated with the glass transition.

In contrast, no transitions were observed in the DSC curve for the pure β-TCP modifier within the studied temperature range.

**Figure 10:**
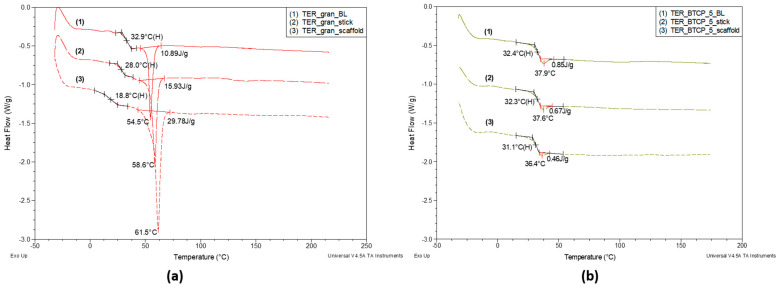
DSC curves for tested terpolymer in melt, stick, and scaffold forms, both pure (**a**) and with 5% β-TCP (**b**), recorded over a temperature range of −30 °C to approx. 180 °C. Measurement conditions are described above, with analysis of glass transition and melting regions.

The thermographic characteristics described above remained consistent despite changes in the sample’s form. Figure 10 compares the DSC curves for the pure terpolymer Figure 10a and the terpolymer with 5% β-TCP Figure 10b in three forms: disks (BL), injection-molded sticks, and 3D-printed scaffolds.

For the pure terpolymer, the glass transition temperature (Tg) decreased significantly and monotonically, from 32.9 °C for the disk to 28.0 °C for the filament and further to 18.8 °C for the scaffold. This phenomenon was accompanied by a progressive increase in the melting peak minimum temperature—indicative of larger average crystallite sizes—from 54.5 °C (melt) to 58.6 °C (filament) and 61.5 °C (scaffold).

Additionally, the melting enthalpy (ΔHm) increased in the same order, from 10.89 J/g for the disk to 15.93 J/g for the filament and, finally, to 29.78 J/g for the scaffold.

For the terpolymer modified with 5% β-TCP (Figure 10b), the trends in the glass transition temperature (Tg) across the three different forms were maintained but significantly less pronounced. The Tg differences between the processing forms amounted to just over 1 °C, which is a stark contrast to the unmodified terpolymer, where ΔTg reached 14.1 °C. Similarly, the minimum temperature difference of the enthalpy relaxation peak was only 1.5 °C. However, the enthalpy of apparent melting changed by as much as 46%. This indicated that the β-TCP addition strongly influenced the terpolymer physical structure, effectively inhibiting crystallization. The β-TCP impact on the amorphous phase formation was minimal, as evidenced by the negligible shift in Tg (0.5 °C) for the TER_gran_BL and TER_β-TCP_5_BL disks, even in the presence of the enthalpy relaxation effect. Moreover, the modifier significantly reduced the material sensitivity to structural changes induced by processing techniques. This was particularly evident in the nearly threefold increase in the melting enthalpy of the unmodified scaffold (29.78 J/g) compared to the unmodified disk (10.89 J/g). The observed regularity may be attributed to the orientation phenomena occurring during the filament formation and, especially, the scaffold printing. This hypothesis was supported by the DSC curve analyses performed on the specific samples over two consecutive heating cycles.

**Figure 11:**
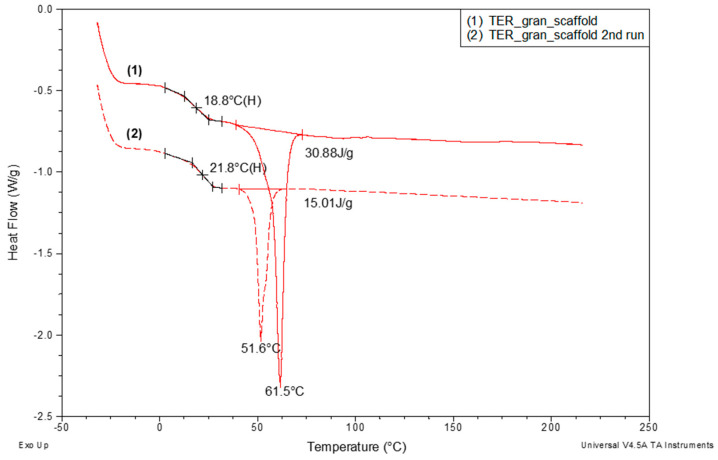
DSC curves for unmodified investigated terpolymer recorded over a temperature range of −30 °C to 225 °C during the first heating run (scaffold) and second heating run (melted scaffold). Measurement conditions are described above, with analysis of glass transition and melting regions.

Figure 11 presents the DSC curves recorded during the TER_gran_scaffold heating. The results revealed that the melting enthalpy (ΔHm) of the crystalline phase in the scaffold—whose structure was significantly influenced by orientation during the printing process—was more than twice as high (30.88 J/g) as the same material’s ΔHm after melting and subsequent cooling (15.01 J/g). This increase was accompanied by a notable rise of nearly 10 °C in the melting peak minimum temperature, which correlated with an increase in the mean crystallite size within the scaffold. Additionally, a threefold increase in the recorded glass transition temperature was observed during this sample reheating.

**Figure 12:**
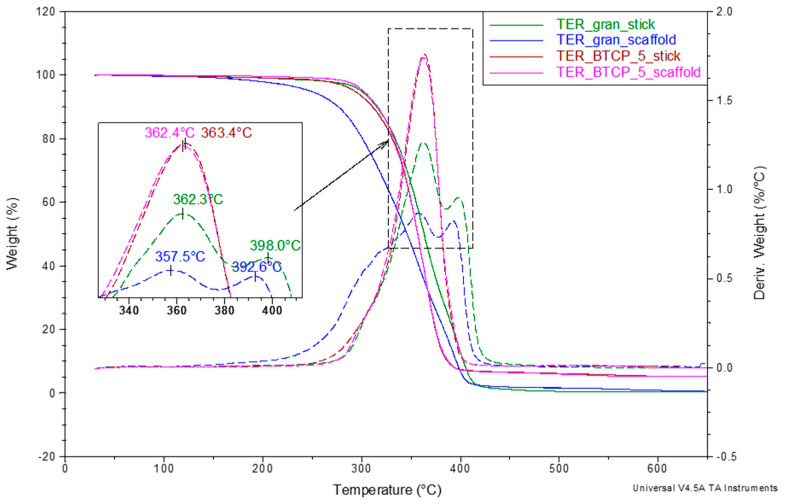
TG and DTG curves of shape memory terpolymer sticks and scaffolds, both unmodified and modified with β-TCP, recorded in heating mode at 20 °C/min under nitrogen purge of 60 mL/min over a 30 to 650 °C temperature range.

The final stage of the thermal analysis involved thermogravimetric studies aimed at evaluating the β-TCP effect on the thermal stability of the developed shape memory terpolymer. The measurements were performed over a wide temperature range (30 to 650 °C) in an inert atmosphere. The results, which are presented as the TG and DTG curves in Figure 12, revealed significant differences in the samples’ thermal decomposition. For the samples containing the β-TCP modifier, the decomposition was single-stage, with the maximum weight loss rate observed at approx. 363 °C. In comparison to the unmodified samples, the decomposition occurred more dynamically and ended at a 20–30 °C cooler temperature. In contrast, the unmodified terpolymer underwent at least a two-step decomposition, with an additional shoulder observed on the DTG curve for the TER_β-TCP_5_scaffold. Notably, the decomposition onset for this sample was approximately 80 °C lower than that of the modified terpolymer scaffold.

This discrepancy suggested that the unmodified terpolymer, subjected to successive technological operations (melting, filament formation, and printing) and corresponding thermal loads, underwent limited depolymerization. Due to this, the macromolecular chains were shortened, which altered their supramolecular structure (as shown in Figure 10) and lowered the decomposition onset temperature. Apparently, the β-TCP addition effectively inhibited the depolymerization process. However, further studies of changes in the average molecular weight are necessary to confirm this hypothesis.

### 3.5. Shape Memory Properties of Terpolymer and β-TCP Modified Materials

**Figure 13:**
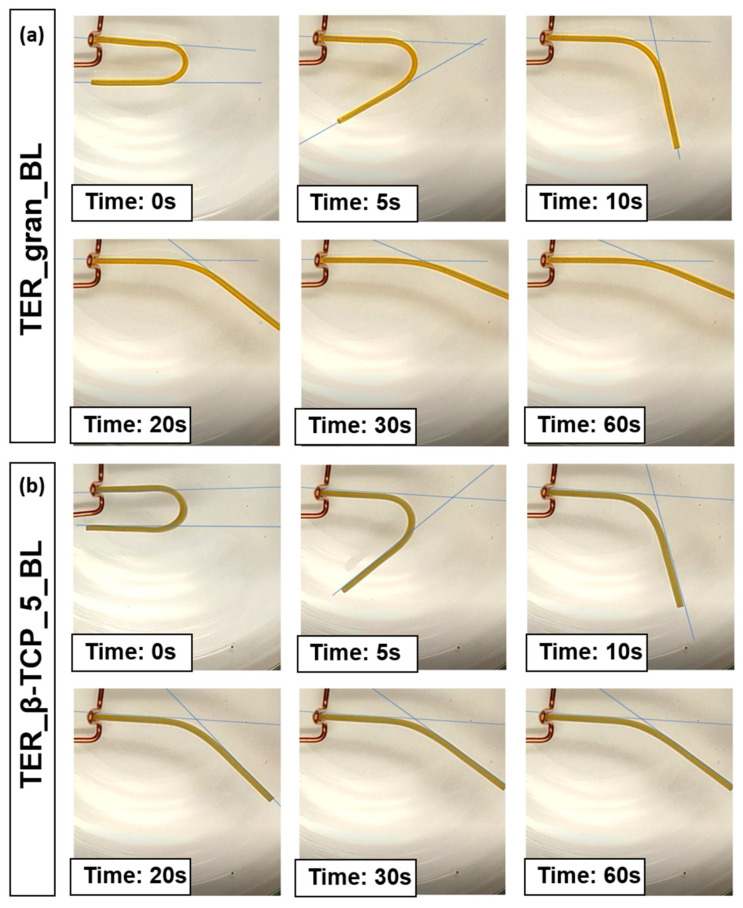
Photographs of samples made from (**a**) TER_gran_BL and (**b**) TER_β-TCP_5_BL alloys during recovery to their original shape.

The recorded sample deformation process was subjected to a frame-by-frame analysis. Based on the observations of the phenomenon, it was determined that deformation readings of the sample would be taken at specific time intervals: t = 0 (immediately after immersing the sample in water), followed by t = 5, 10, 15, 20, 25, 30, 40, 50, and 60 s. The change in the sample’s bending angle relative to its initial bending angle, expressed as a percentage, was employed to assess the sample’s ability to return to its original shape. Figure 13 presents the results of the frame-by-frame analysis of the selected samples at specific time intervals.

**Figure 14:**
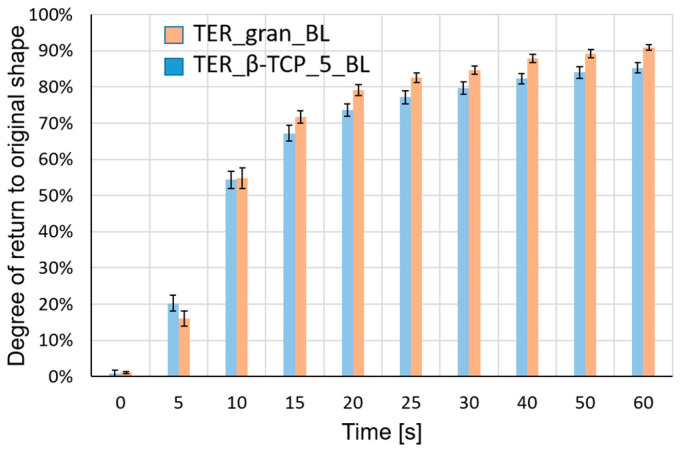
Average recovery degree of samples with standard deviation. Shape memory studies demonstrated enhanced ability to return to original shape in samples made from pure terpolymer (TER_gran_BL) compared to samples made from terpolymer modified with additive (TER_β-TCP_5_BL). The error bars represent the standard deviation (SD) of the measurements at each time point. After 20 s, the differences between the two materials were statistically significant.

Figure 14 shows a summary of the results of the measurements of the samples’ ability to return to their original shapes.

### 3.6. Cell Culture Results

**Figure 15:**
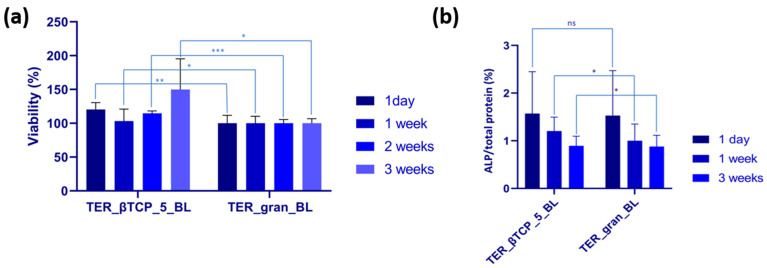
(**a**) MTT assay showing Saos-2 cell viability, expressed as control percentage. Data presented as mean ± SD (n = 3). Statistical analysis performed using *t*-tests, with the following significance levels: nonsignificant (ns) at *p* > 0.05, significant at *p* < 0.05 (*), *p* < 0.01 (**), and *p* < 0.001 (***). (**b**) ALP activity after incubation of Saos-2 cells with tested samples. Data shown as mean ± SD (n = 3). Statistical analysis performed using *t*-tests. Nonsignificant (ns) at *p* > 0.05 and significant at *p* < 0.05 (*).

Saos-2 cells were incubated with the modified materials for 1 day, 1 week, 2 weeks, and 3 weeks at 37 °C in 5% CO_2_. The material modified with β-tricalcium phosphate (TER_βTCP_5_BL) was compared to the plain material (TER_gran_BL), which served as the control. The cells in contact with TER_βTCP_5_BL exhibited higher viability than those cultivated on TER_gran_BL throughout the whole incubation period, with significance at *p* < 0.01 for 1 day, *p* < 0.05 for 1 week, *p* < 0.001 for 2 weeks, and *p* < 0.05 for 3 weeks (Figure 15a). The highest viability was observed after 3 weeks of incubation (149.82%). The ALP, a key enzyme involved in mineralization and bone development, was evaluated in osteoblast-like cells (Saos-2) cultivated on the samples for periods ranging from 1 day to 3 weeks. After 1 day of incubation, no significant differences in the ALP activity were observed (Figure 15b). However, after 1 week and 3 weeks, the cells grown on TER_βTCP_5_PL exhibited higher ALP activity compared to those on TER_gran_BL.

**Figure 16:**
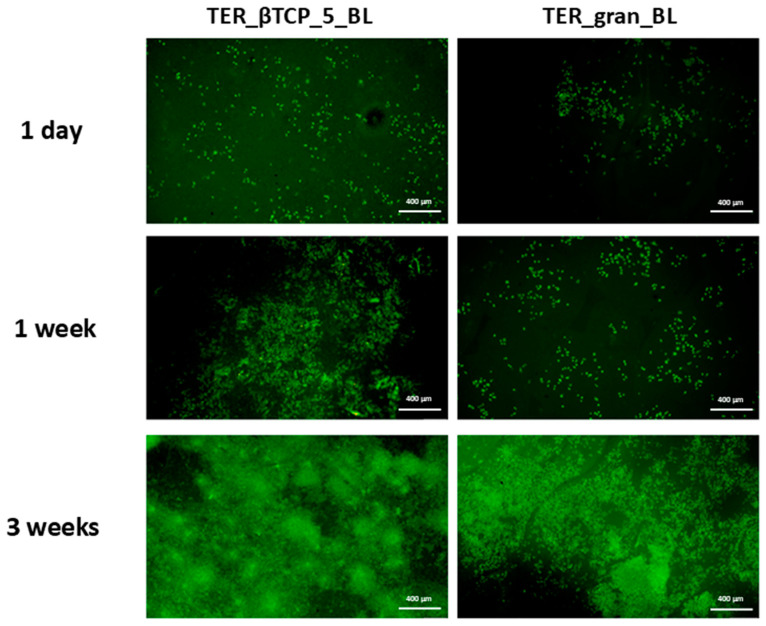
Fluorescence microscopy images of cells cultured on β-tricalcium phosphate-modified materials and unmodified materials (TER_gran_BL) after 1 day, 1 week, and 3 weeks of incubation at 4× magnification. Cells stained with acridine orange, highlighting viable cells in green (n = 3).

Figure 16 shows the representative fluorescence microscopy images of the cells cultured on the materials after 1 day, 1 week, and 3 weeks. A time-dependent increase in the number of living cells (stained green) was observed on both TER_βTCP_5_BL and TER_gran_BL.

**Figure 17:**
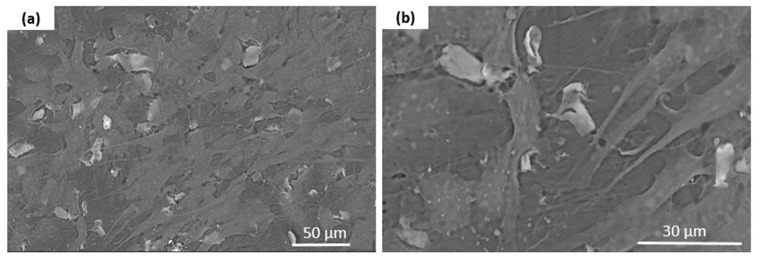
SEM images (**a**,**b**) showing cells growing on a β-TCP-modified terpolymer.

In Figure 17, SEM images show cells growing on a β-TCP-modified terpolymer. The cells exhibit a spread morphology with visible filopodia, indicating good adhesion and interaction with the substrate. Mineralized deposits are also evident, demonstrating calcium and phosphate ion release from β-TCP, which supports mineralization and bone-like structure formation.

## 4. Discussion

Polyglycolide (PGA) copolymers with poly(ε-caprolactone) (PCL) or polylactide (PLA) have gained considerable attention in biomedical applications due to their tunable mechanical properties, which can be adjusted by varying the monomer composition [23]. Extensive research has focused on the synthesis and development of terpolymers for medical applications [24]. In our study, we utilized a terpolymer composition of L-lactide/glycolide/ε-caprolactone (69/16/15), which was further modified with β-tricalcium phosphate (β-TCP) to enhance its bioactivity. As is already known, β-TCP incorporation improves cell adhesion, proliferation, and osteogenic differentiation, making it a promising modifier for biomedical materials [13].

The developed composite material, based on a shape memory polymer (SMP) matrix with β-TCP, was designed to maintain shape memory effects and self-deformation properties suitable for 4D printing. The composite exhibited excellent processability with a 3D printer, biocompatibility, and biodegradability. Additionally, the shape memory performance of the terpolymer was remarkable, achieving a shape recovery ratio exceeding 80% at body temperature. After 1 min, the pure terpolymer samples demonstrated slightly higher recovery rates (~90%) when compared to the β-TCP-modified samples (~80%), confirming the modifier influence on the shape memory properties.

The shape memory properties demonstrated in Figure 13 and Figure 14 have significant implications for the functionality of implants. Senatov et al. demonstrated the potential of shape recovery for self-fitting small bone defect implants by achieving 98% recovery in polylactide/15 wt% hydroxyapatite scaffolds at 80 °C [25]. Building on similar principles, Zarek et al. fabricated a thermally actuated tracheal stent, which expanded safely within the trachea [26]. Other researchers have explored temperature-responsive hydrogels with reversible transformations driven by differential shrinkage/swelling [27]. Additionally, Hippler et al. developed programmable poly(N-isopropylacrylamide)-based valves with precise actuation, enabling applications in fields like soft robotics, microfluidics, and biosciences [28]. The ability of the material to recover its original shape upon exposure to specific thermal triggers allows implants to be designed in a compact form for easier insertion [29]. Once implanted, the material can expand or reshape itself to conform to complex anatomical structures, enhancing stability and fit. This adaptability not only facilitates minimally invasive surgical procedures, thereby reducing tissue trauma and recovery time, but also allows the implant to dynamically respond to changes in body temperature and surrounding conditions [30]. Furthermore, these properties can improve the integration of the implant with surrounding tissues, promoting better biological acceptance and mechanical support, ultimately leading to improved patient outcomes.

The microscopic and spectroscopic analyses proved the successful integration of β-TCP within the terpolymer matrix. The SEM and EDS analyses showed evenly distributed β-TCP inclusions, with minor agglomeration in the scaffold samples. The FTIR spectroscopy examinations further confirmed the effective β-TCP chemical integration into the polymer. The DSC calorimetric studies revealed that β-TCP and the processing methods significantly affected the nanostructure, influencing both the crystalline and amorphous phases. The TGA analysis highlighted a potential risk of limited depolymerization under thermal loads. However, the β-TCP modifier mitigated this risk by promoting crystallization stability. The biological evaluations reinforced the benefits of β-TCP modification. The MTT assay results showed significantly enhanced Saos-2 cell viability on the β-TCP-modified materials (TER_βTCP_5_BL) compared to the control terpolymer (TER_gran_BL). This effect was most pronounced after 2 weeks, indicating that β-TCP supported the cell proliferation. Fluorescence microscopy demonstrated a time-dependent increase in the viable cell density, with the β-TCP-modified materials consistently outperforming the control. The alkaline phosphatase (ALP) activity, which is a critical marker for mineralization and bone development, was significantly elevated in the β-TCP-modified samples after 1 and 3 weeks of incubation, underscoring the material osteogenic potential. These findings aligned with previous studies, e.g., Lu et al., which highlighted the benefits of β-TCP in scaffolds for promoting osteogenic differentiation and bone tissue regeneration [31].

The SEM images depict cells growing on a β-TCP-modified terpolymer, highlighting promising biocompatibility, cell–material interactions, and evidence of mineralization. The cells exhibit a spread and flattened morphology, indicating good adhesion and adaptation to the substrate, with visible thin, thread-like projections (filopodia) suggesting active exploration and anchorage. The β-TCP modification enhances surface roughness and porosity, facilitating cell attachment, migration, and interaction with the material. Furthermore, mineralized deposits are observed on the surface, suggesting active calcium and phosphate ion release from β-TCP, which not only promotes cellular activity but also supports the formation of bone-like structures. β-TCP-modified terpolymer provides an ideal environment for cell adhesion, proliferation, differentiation, and mineralization, making it highly suitable for bone tissue engineering applications.

The shape memory terpolymer developed in this study offers significant promise for medical implant applications, particularly in the regeneration of cartilage and subchondral bone. Its ability to recover shape upon exposure to specific environmental stimuli could allow for the production of scaffolds that adapt to complex anatomical structures. Such scaffolds could be used in surgeries where the implants need to fit irregularly shaped tissue defects. Additionally, the biocompatibility of the material is essential for ensuring that the scaffold integrates well with the surrounding tissue, promoting healing and tissue regeneration.

While the shape memory terpolymer presents a novel approach to tissue engineering, its limited elongation and mechanical properties must be carefully considered for load-bearing applications. For scaffolds used in bone regeneration, the material’s high tensile strength and dimensional stability are advantageous, as they can withstand mechanical stresses. However, its lower elongation, compared to more flexible materials like polycaprolactone (PCL), could limit its use in applications requiring significant deformation. Nonetheless, this feature may be beneficial in certain applications where maintaining structural integrity is more critical than flexibility, such as in the repair of subchondral bone defects.

The successful use of materials in cartilage and bone replacements depends on their ability to meet both mechanical and biological requirements. Mechanical properties such as tensile strength, elasticity, and stiffness must align with the physiological conditions of the tissue being replaced. In the case of bone scaffolds, the terpolymer’s high tensile strength, combined with its ability to maintain structural integrity, positions it as a promising candidate for load-bearing applications. In terms of biological compatibility, the material’s biocompatibility, as demonstrated in our tests, ensures it can support cell growth and tissue integration, which are critical factors for successful implant integration and long-term function.

## 5. Conclusions

The aim of this study was to evaluate the effect of β-tricalcium phosphate (β-TCP) modification on the structural, mechanical, thermal, and functional properties of a shape memory terpolymer, with a focus on its potential application in 4D printing technology and medical implant production. The integration of β-TCP into the filament matrix and scaffolds was confirmed through microscopic (SEM, stereoscopic) and spectroscopic (FTIR, EDS) analyses. The FTIR spectra indicated the successful incorporation of β-TCP into the polymer. The differential scanning calorimetry (DSC) analysis revealed changes in thermal transitions, suggesting improved processing properties, particularly for 3D printing, due to β-TCP modification. The thermal stability was enhanced as β-TCP delayed the polymer matrix depolymerization. The shape memory studies demonstrated effective recovery in both the modified and unmodified samples, although β-TCP slightly reduced the recovery performance. The in vitro cell culture studies showed that the β-TCP-modified terpolymer significantly increased the cell viability values and the alkaline phosphatase (ALP) activity after 3 weeks. The β-TCP-modified terpolymer can be tailored for applications where partial shape recovery is acceptable, such as bone scaffolds or implants designed to promote osteointegration.

The integration of “time” as the fourth dimension in 4D printing introduces dynamic functionality, enabling intricate structures with customizable forms for advanced tissue engineering applications. Current shape transformations in 4D printing remain basic, requiring improved spatiotemporal control, enhanced mechanical robustness, and optimized stimuli-responsive materials.

## Figures and Tables

**Table 1:** Parameters of the injection molding process for filament sticks and samples for mechanical testing (dog bone shape).

Parameters	Filament Sticks		Dog Bone-Shaped Samples	
	TER_gran	TER_β-TCP	TER_gran	PCL
Plastification temperature [°C]	125	135	125	160
Chamber temperature [°C]	130	132	130	151
Nozzle temperature [°C]	140	131	140	165
Injection size [mm]	18	18	24	24
Cooling time [s]	28	28	60	60
First injection pressure [bar]	113	107	113	130
Time of the first injection pressure [s]	1.9	2.0	11	11
Second injection pressure [bar]	60	60	90	90
Time of the second injection pressure [s]	5.3	5.3	3.5	3.5

**Table 2:** Mechanical properties of pure terpolymer and longitudinal wave velocity determined from ultrasonic testing.

Type of Sample	Young’s Modulus [MPa]	Tensile Strength [MPa]	Strain at Break [%]	V_L_^1^ [m/s]
Terpolymer Polycaprolactone	1492.4 ± 129.1 407 ± 20	45.95 ± 3.25 31.95 ± 1.95	3.36 ± 0.35 658.99 ±46.09	2312.5 ± 13.8 1995 ± 10.5

^1^ V_L_—longitudinal wave velocity.

## Data Availability

The raw/processed data required to reproduce these findings cannot be shared at this time, as the data are also part of an ongoing study.
